# The Emergence of Japanese Encephalitis Virus in Australia in 2022: Existing Knowledge of Mosquito Vectors

**DOI:** 10.3390/v14061208

**Published:** 2022-06-02

**Authors:** Andrew F. van den Hurk, Eloise Skinner, Scott A. Ritchie, John S. Mackenzie

**Affiliations:** 1 Public Health Virology, Forensic and Scientific Services, Department of Health, Queensland Government, Brisbane, QLD 4108, Australia; 2 Centre for Planetary Health and Food Security, Griffith University, Gold Coast, QLD 4222, Australia; ebskinn@stanford.edu; 3 Department of Biology, Stanford University, Stanford, CA 94305, USA; 4 College of Public Health and Medical and Veterinary Sciences, James Cook University, Cairns, QLD 4878, Australia; scott.ritchie@jcu.edu.au; 5 Faculty of Health Sciences, Curtin University, Bentley, WA 6102, Australia; j.mackenzie@curtin.edu.au; 6 School of Chemistry and Molecular Biosciences, The University of Queensland, St. Lucia, QLD 4072, Australia

**Keywords:** Japanese encephalitis virus, Australia, mosquitoes, virus detection, vector competence, host feeding patterns

## Abstract

In early 2022, the Japanese encephalitis virus (JEV) was identified as the cause of stillborn and mummified piglets in pig farms in southeastern Australia. Human cases and additional pig farms with infected piglets were subsequently identified across a widespread area encompassing four states. To inform surveillance and control programs, we synthesized existing information on Australian vectors of JEV, much of which was generated in response to incursions of JEV into the northern state of Queensland between 1995 and 2005. Members of the *Culex sitiens* subgroup, particularly *Culex annulirostris*, should be considered the primary vectors of JEV in Australia, as they yielded >87% of field detections of JEV, were highly efficient laboratory vectors of the virus, readily fed on pigs and birds (the key amplifying hosts of the virus) when they were available, and are widespread and often occur in large populations. Three introduced species, *Culex quinquefasciatus*, *Culex gelidus* and *Culex tritaeniorhynchus* may also serve as vectors, but more information on their geographical distribution, abundance and bionomics in the Australian context is required. Mosquitoes from other genera, such as *Aedes* and *Verrallina*, whilst considered relatively poor vectors, could play a regional or supplemental role in transmission, especially facilitating vertical transmission as a virus overwintering mechanism. Additional factors that could impact JEV transmission, including mosquito survival, dispersal and genetics, are also discussed. Possible directions for investigation are provided, especially in the context of the virus emerging in a region with different mosquito fauna and environmental drivers than northern Australia.

## 1. Introduction

The Japanese encephalitis virus (JEV) is the leading cause of encephalitis in Southeast Asia and is responsible for an estimated 68,000–100,000 cases per year, although this is likely an underestimate [1,2]. It is a positive-sense RNA virus that was classified into five genotypes whose distributions vary due to climate and other environmental factors [3,4,5]. The virus exists in an enzootic transmission cycle involving ardeid wading birds and mosquitoes [6,7]. Pigs are also amplifying hosts and are often involved in the epizootic amplification of the virus, which results in spillover to humans [8,9]. Whilst humans and horses can develop fatal encephalitis, they are considered dead-end hosts of the virus because they do not produce sufficient viremia levels to infect mosquitoes [10]. Pigs can also develop a disease that is characterized by reduced sperm production in boars and fetal abortion in infected sows (reviewed in [11]).

**Figure 1:**
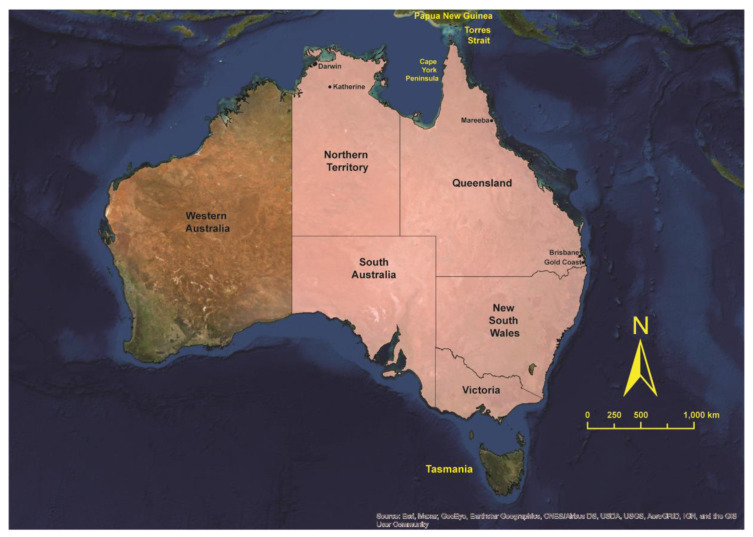
Map of Australia and Papua New Guinea showing locations discussed in the text. The states and territories that are shaded pink had reported human cases of Japanese encephalitis, piggeries with infected pigs and/or positive feral pigs in 2022.

JEV, like other important flaviviruses, such as West Nile virus (WNV) and dengue viruses, shows a strong propensity to spread into new geographic areas [12]. In 1995, JEV emerged in the Australasian zoogeographical region when an outbreak of genotype 2 occurred on the islands of the Torres Strait of northern Australia (Figure 1; [13]). The 1995 outbreak and another, more geographically widespread outbreak in 1998 [14], resulted in five human cases, two of which were fatal. The virus was subsequently identified in the Western Province of Papua New Guinea [15,16], and it was thought that this was the source of incursions into Australia [13,17]. A sentinel pig program detected JEV activity every year (except 1999) in the Torres Strait between 1995 and 2005 [18,19], although identification of only genotype 1 viruses from the year 2000 onward suggested that a separate incursion of the virus into the region had occurred [20]. Activity in 1998 and 2004 spread onto Cape York Peninsula, but there was no evidence that the virus had become established in natural transmission cycles on the mainland [14,21], despite ecological conditions potentially being suitable for its establishment [22]. The status of JEV in northern Australia had become less clear, as most forms of targeted surveillance were gradually phased out after 2011 [19].

In early 2021, JEV was diagnosed in a resident of the Northern Territory [23], signifying another incursion of the virus into Australia and the first confirmed evidence of virus activity since 2005 [24]. In February 2022, JEV was identified as the virus responsible for an increased number of stillbirths, mummified fetuses and piglets with neurological disease at commercial pig farms in three eastern Australian states [25]. As of 5 May 2022, a total of 70 piggeries across four states had been affected and the virus was detected in feral pigs in the Northern Territory [26]. The first human cases of encephalitis caused by JEV infection were reported in early March 2022 and, as of 25 May 2022, there were 30 confirmed cases, with five deaths, reported across four states [27]. The unprecedented emergence of JEV across such a wide geographical area of southeastern Australia has necessitated a multi-sectoral response aimed at determining the distribution of the virus, the potential for its permanent establishment, ongoing human and animal disease risk, and options for sustainable control of virus transmission.

To inform a “One Health” response to the emergence of JEV, we provide a synthesis of the existing knowledge of Australian mosquito vectors of the virus. To achieve this, we initially employed criteria originally proposed by Reeves [28] and applied by Turell et al. [29] to incriminate different mosquito species in the transmission of WNV in North America. These criteria include (a) detection of the virus in field-collected mosquitoes, (b) a demonstrated ability of the mosquito species to become infected with and transmit the virus, and (c) a demonstrated association between the mosquito species and vertebrate hosts of the virus. Other factors not intrinsically linked to the direct interaction with the virus and a vertebrate host, but which can impact the role a mosquito species plays in transmission cycles, such as mosquito genetics, survival, dispersal, abundance, and geographical and temporal distributions, are also discussed.

## 2. Incrimination of Australian Mosquitoes as Vectors of JEV

### 2.1. Detection of JEV in Field Populations of Mosquitoes

In response to recognized JEV incursions or to further characterize the distribution of the virus in the region, mosquitoes were collected between 1995 and 2005 from several locations in northern Australia and Papua New Guinea. Mosquitoes were predominately collected using Centers for Disease Control miniature light traps baited with CO_2_, which were often supplemented with 1-octen-3-ol. Gravid traps and various propane or solar-powered traps were also deployed on occasion in the Torres Strait [24,30]. Mosquitoes were pooled by species or another taxonomic group, which included damaged specimens and mosquitoes that were morphologically indistinguishable (i.e., the *Anopheles farauti* species complex; [31]). Of note, difficulty in reliably separating *Culex annulirostris*, *Cx. palpalis* and *Cx. sitiens* [32] meant that for some investigations, they were not identified at the species level and were just referred to as *Cx. sitiens* subgroup mosquitoes. In some studies, a subsample of mosquitoes or the species composition of positive pools was identified using isoenzyme analysis or molecular assays to indicate the species present [33,34]. JEV was detected using a cell culture immunoassay [35]), or viral RNA was detected using real-time reverse transcriptase (RT) PCR [36].

A total of 540,882 mosquitoes were processed from northern Australia and Papua New Guinea between 1995 and 2000 during periods where JEV was detected from at least one pool of mosquitoes (Supplementary Table S1). This allowed for an assessment of which species or taxonomic groups were infected whilst the virus was actively circulating in a given location. From at least 38 species across 12 genera processed, over 60% of mosquitoes belonged to the genus *Culex*, from which, 92% were members of the *Cx. sitiens* subgroup, predominately *Cx. annulirostris*. Of the remaining genera, *Aedes* and *Verrallina* represented 18% and 11% of the mosquitoes processed, respectively.

Between 1995 and 2000, JEV was isolated from 56 pools of mosquitoes, with members of the *Culex* genus accounting for all but one of the isolates (Table 1). Fifty-four of the isolates were obtained from members of the *Cx. sitiens* subgroup, whilst single isolates were obtained from *Ae. vigilax* and *Cx. gelidus*. Most isolates (93%) were obtained from mosquitoes collected from Badu Island, with all but one from collection trips undertaken in 1995 and 1998. Despite collections from Papua New Guinea comprising 73% of the *Cx. sitiens* subgroup mosquitoes processed, only three isolates were obtained, although, unlike Badu, there was no other evidence of epizootic activity occurring when the collections were undertaken.

Based on their role as the primary vectors of JEV, surveillance of the virus in northern Australia conducted between 2003 and 2005 focused predominately on *Culex* spp. During this period, 33,931 *Culex* mosquitoes were processed from Badu Island, from which, JEV RNA was detected in 23 pools (Table 1). Viral RNA was also detected in three pools of *Culex* mosquitoes collected from the nearby Moa Island. All but five of these detections were in pools of *Cx. sitiens* subgroup mosquitoes, with viral RNA detected in four pools of *Cx. gelidus* and one pool of *Cx. bitaeniorhynchus*, with the latter consisting of two mosquitoes collected in a gravid trap. One of the detections in *Cx. gelidus* and the *Cx. bitaeniorhynchus* detection are reported here for the first time and are the result of further analysis of data presented in van den Hurk et al. [30]. Between 2002 and 2005, a new trapping strategy was deployed to remotely collect mosquitoes over several weeks, which were then processed in large pool sizes using real-time RT-PCR [24]. In 2002, no attempt was made to identify the mosquitoes from the traps that contained approximately 178,000 mosquitoes; therefore, the taxonomic group composition of the collections from which JEV RNA was detected in three pools is unknown. In 2004, 23,144 *Culex* spp. were processed from mainland Australia, from which, JEV was detected in a single pool of *Cx. sitiens* subgroup mosquitoes [21].

Any or a combination of the three Australian members of the *Cx. sitiens* subgroup could have comprised the species within a JEV-positive pool. *Cx. annulirostris* were reliably identified during the original field studies on Badu [37] and molecular analysis suggested it was the dominant species during other collection trips [21,40]. Although no isolates have been obtained from mosquitoes identified exclusively as *Cx. sitiens* in Australia, possibly reflecting the low numbers of mosquitoes of this species processed in the region, this species has yielded isolates in Southeast Asia [42,43,44]. The isolation of JEV from Lake Murray in PNG in 1997 was from pools of *Cx. sitiens* subgroup mosquitoes, of which, a significant number were subsequently shown to be *Cx. palpalis* [15,33,34].

### 2.2. Intrinsic Ability of Australian Mosquitoes to Become Infected with and Transmit JEV

**Figure 2:**
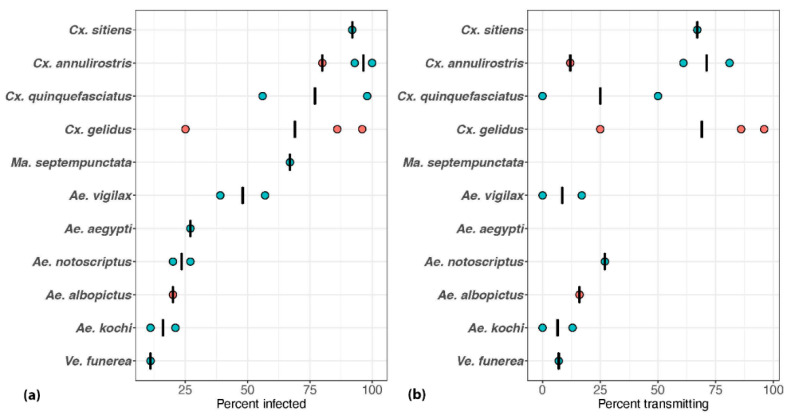
Results of the vector competence experiments conducted to assess the ability of Australian mosquito species to (**a**) become infected with and (**b**) transmit the Japanese encephalitis virus. Mosquitoes were allowed to feed on an infectious blood meal containing 10^6−7^ infectious units of virus per milliliter and tested at days 10–14 post-exposure. The orange and blue circles represent experiments conducted with genotype 1 and genotype 2 viruses, respectively, and the bars represent the means of data derived from separate virus exposures. Previously published studies [45,46,47,48] provided the source data. A minimum of five mosquitoes was required for inclusion in the analysis. *Mansonia septempunctata* and *Ae. aegypti* were not tested for their ability to transmit the virus.

Whilst detection of the virus or viral RNA in field populations can implicate species in arbovirus transmission cycles, laboratory-based vector competence experiments provide evidence that the virus can indeed replicate and be transmitted by implicated species. A total of 17 Australian mosquito species were assessed for their ability to become infected with Australian strains of JEV, with 10 of these species also tested for their ability to transmit the virus [45,46,47,48]. The highest infection and transmission rates were observed in the *Culex* spp., particularly *Cx. annulirostris*, *Cx. gelidus* and *Cx. sitiens*, with the proportion infected with and transmitting the virus for these three species being >90% and >65%, respectively (Figure 2). The proportion of other genera infected was mostly <50% and <30% of individual mosquitoes tested transmitted the virus. A preliminary investigation of a Northern Territory population of *Cx. palpalis* revealed that this species was able to transmit the genotype 3 Nakayama strain of JEV to suckling mice (L. Melville, unpublished data).

Vector competence for JEV appears to vary within mosquito species, as well as between virus genotypes tested in mosquito species of the same origin. For instance, *Cx. annulirostris* was a highly efficient laboratory vector of the genotype 2 JEV, which contrasted with its relatively low vector competence for the genotype 1 JEV that appeared to have become the dominant genotype in northern Australia [45,48]. There were also differences in vector competence of two populations of *Cx. quinquefasciatus* for genotype 2 JEV, with a colony of Gold Coast (southern Queensland) origin having a 50% transmission rate, whilst none of the 16 field-derived mosquitoes from Mareeba (north Queensland) transmitted the virus [48]. Similar differences in vector competence between populations were observed for JEV in *Cx. tritaeniorhynchus* in Southeast Asia [49,50], and for Murray Valley encephalitis (MVEV) and West Nile virus (Kunjin subtype; WNV_KUN_) in *Cx. annulirostris* in Australia [51]. It is also important to note that variation in the vector competence could be due to differences in the experimental methodology, such as the use of colony versus field-collected mosquitoes, the method used to assess transmission or the assay used to detect the virus [52].

The extrinsic incubation period (EIP) of the virus is the time taken from when the mosquito imbibes the virus in an infectious blood meal to when it can transmit it via the saliva during a subsequent blood feed. The EIP plays a critical role in transmission dynamics because if it is short, then a much higher proportion of mosquitoes will survive to transmit the virus. At 28 °C, both *Cx. annulirostris* and *Cx. sitiens* were able to transmit the virus to a small proportion of mice on day 7 post-exposure (the earliest day tested), but the highest rates of transmission did not occur until day 14 post-exposure [48]. These results are similar to those reported for *Cx. tritaeniorhynchus* incubated at 28 °C and tested with an in vitro method of measuring transmission [53]. This latter study found that the EIP increased considerably when mosquitoes were incubated at 20 °C and transmission did not occur until day 20 post-exposure.

### 2.3. Host-Feeding Patterns of Mosquitoes Implicated as JEV Vectors

**Figure 3:**
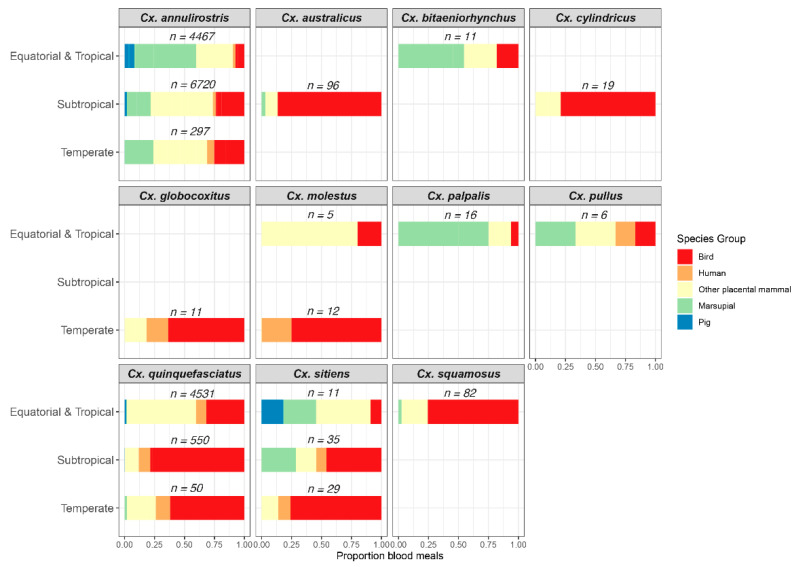
Identification of the vertebrate source of blood meals of Australian species of *Culex*, with a focus on animal groups of importance to Japanese encephalitis virus transmission cycles. Source data were from previously published studies [56,57,58,59,60,61,62,63,64].

Analysis of host-feeding patterns provides important data on the proportion of blood meals originating from vertebrate hosts of the virus, which for JEV are birds and pigs. In these studies, blood-engorged mosquitoes were collected from the field and the vertebrate source of the blood in the abdomen was identified using either serological or molecular assays [54,55]. We synthesized data from nine studies on the host-feeding patterns of Australian mosquitoes [56,57,58,59,60,61,62,63,64], with a particular focus on *Culex* spp. (for which ≥5 blood meals had been reported). A total of 16,948 blood meals from 11 mosquito species were identified and patterns were summarized across three climatic regions (Figure 3).

Across all regions, *Cx. annulirostris* was an opportunistic feeder, taking blood meals from a diversity of vertebrate groups. Overall, pigs accounted for only a small proportion of blood meals identified. The paucity of blood meals originating from pigs in some sub-tropical and temperate areas could represent the fact that pigs were not present in the collection areas or pig antisera were not included in the panel of antisera used in the serological assays. In equatorial and tropical areas, pig-feeding proportions >30% were identified only where domestic pigs were housed or in locations where feral pigs congregated, such as rubbish dumps [58,64]. Instead, other mammals, particularly macropods, comprised the greater proportion of blood meals identified [64,65]. As wallabies and kangaroos are poor amplifying hosts of the virus [66], it was hypothesized that high feeding rates on macropods may impede the transmission cycle [67]. Whilst *Cx. annulirostris* did feed on birds, it was only in temperate regions where birds accounted for >20% of the blood meals identified. Interestingly, by employing DNA sequence analysis, Jansen et al. [59] identified *Cx. annulirostris* blood meals that had originated from the Nankeen night heron *Nycticorax caledonicus*, one of the ardeid bird species suspected to play a role in JEV transmission in Australia. Across all regions, <10% of *Cx. annulirostris* blood meals originated from humans.

Of the other *Culex* spp. implicated in JEV transmission in Australia, birds accounted for >70% of blood meals identified in some regions. Pigs accounted for almost 20% of blood meals identified from *Cx. sitiens* in the tropical region, but this may have reflected the small number of blood meals tested rather than any specific host-feeding pattern. Except for the temperate populations, *Cx. quinquefasciatus* obtained >50% of its blood meals from mammals contrasting with its perception as an ornithophilic species. Only a small proportion of blood meals from this species, as well as *Cx. sitiens*, originated from humans.

## 3. Australian Vectors of JEV: Reviewing the Evidence

### 3.1. Mosquitoes Implicated from Field and/or Laboratory Studies

Based on the number of field detections, vector competence and host-feeding patterns [28], members of the *Cx. sitiens* subgroup should be considered the major vectors of JEV in Australia. Given that *Cx. annulirostris* is a widely distributed species in Australia, is a highly efficient laboratory vector of JEV and readily feeds on pigs and birds, when available, this species should be considered the primary Australian vector of JEV. This status is not surprising given that it is considered the biological equivalent of *Cx. tritaeniorhynchus* and *Cx. tarsalis*, which are two species that are major vectors of encephalitogenic flaviviruses in their respective regions [68]. The high infection and transmission rates observed in *Cx. sitiens* in the vector competence experiments, coupled with it yielding isolates of the virus in Asia, suggest that this species could act as a vector of JEV, particularly in coastal regions of Australia. Although *Cx. palpalis* potentially yielded isolates in PNG and was able to transmit the virus in the laboratory, more information on its geographical distribution, biology and interaction with amplifying hosts is required to clarify its role in JEV transmission.

There is no doubt that other Australian species of *Culex* could potentially play a role in JEV transmission cycles. Of these, Australian populations of *Cx. gelidus* were highly efficient laboratory vectors of JEV and the virus was detected in this species collected from northern Queensland, despite relatively small numbers being processed. The results of the vector competence experiments suggest that *Cx. quinquefasciatus* could serve as a vector, especially considering that large populations of this species can be associated with effluent ponds and other larval habitats with high organic content. However, only low numbers of *Cx. quinquefasciatus* have been processed to detect the virus, as they are not readily collected in CO_2_-baited light traps and gravid traps were only occasionally deployed to sample them during the JEV investigations of the 1990s and 2000s. Despite only low numbers being processed, the detection in *Cx. bitaeniorhynchus* indicates that Australian populations of this species can become infected with JEV, but their ability to transmit the virus has not been assessed. All three species have been implicated as JEV vectors in Southeast Asia and the Indian subcontinent [42,69,70,71,72], and have host-feeding patterns that bring them into contact with amplifying hosts (Figure 3; [64]); therefore, they should be considered potential vectors of the virus in Australia.

The status of other mosquito genera as vectors of JEV in Australia is less defined and it could be that they may play only a regional or supplemental role in transmission. The > 50% infection and dissemination rates observed in the *Mansonia* spp., coupled with the virus being isolated from members of this genus in India [73], suggest that they could also play a role in transmission. Within the *Aedes* genus, both *Ae. vigilax* and *Ae. notoscriptus* were relatively inefficient laboratory vectors of JEV when compared with *Culex* spp. Although the virus was isolated from *Ae. vigilax*, the minimum infection rate was considerably lower than that observed in the *Cx. annulirostris* that were collected at the same time [38]. The lower vector competence of *Ae. vigilax* and *Ae. notoscriptus* could be offset by their widespread distribution in Australia and high relative abundance when conditions are suitable [74,75]. Other *Aedes* spp. (i.e., *Ae. kochi* and *Ae. culiciformis*) and *Verrallina* spp. (i.e., *Ve. carmenti* and *Ve. funerea*) were incompetent laboratory vectors and/or yielded no field isolates despite 10,000 s of mosquitoes being collected at the same time that multiple isolates were obtained from *Cx. sitiens* subgroup mosquitoes. Perhaps the key role that *Aedes* spp. will play in transmission cycles is virus maintenance through the vertical transmission to progeny via desiccation-resistant eggs [76]. However, in the only experiment assessing this in Australian mosquitoes, there was no evidence that 14 infected *Ae. vigilax* transmitted the virus to their progeny [48].

### 3.2. Potential Australian Vectors

Whilst incriminated vectors, such as *Cx. annulirostris*, *Cx. palpalis*, *Cx. quinquefasciatus* and *Cx. sitiens*, occur in sub-tropical and temperate Australia, there are other species in southern latitudes that could also play an important role in JEV transmission. Potential vectors of JEV would include *Culex* spp., those that have yielded isolates in Southeast Asia or those that have been associated with closely related flaviviruses. For instance, MVEV and WNV_KUN_ were isolated from *Culex australicus*, which is susceptible to both viruses in the laboratory [77,78,79,80]. Likely introduced into Australia in the 1940s [81], *Culex molestus* is a competent laboratory vector of JEV [82]. Furthermore, both species readily feed on birds (Figure 3) and can be locally abundant [83]. There are also some *Aedes* spp., such as *Aedes vittiger* and *Aedes sagax*, that are susceptible to MVEV infection in the laboratory [79,84] and whose populations in southern regions can reach very high levels following flooding rains [83]. If susceptible to JEV infection, then these mosquitoes could provide a mechanism of virus overwintering, similar to other species in the genus.

## 4. Other Factors That Impact the Role of Mosquitoes in JEV Transmission Cycles

The intrinsic ability for a mosquito to become infected with and transmit an arbovirus, coupled with evidence of infection in the field and interaction with the vertebrate host of the virus, are obviously important criteria for conferring vector status. However, there are numerous other factors that can impact the role that different mosquito species play in transmission cycles. Using studies focused on *Cx. annulirostris* but including other species where appropriate, we briefly discuss some of these factors and how they could influence the establishment of JEV in Australia.

### 4.1. Survival

The ability of a mosquito species to survive the EIP of the virus is critical in assessing what role a species can play in transmission. Although *Cx. annulirostris* could transmit JEV as early as day 7 post-exposure, in modeling the transmission dynamics of JEV in Japan, Wada [85] considered that the time from when *Cx. tritaeniorhynchus* imbibed an infectious blood meal to being able to transmit the virus was 10 days. Similarly, in analyzing the transmission dynamics of MVEV, Kay et al. [86] considered that mosquitoes did not become epidemiologically important until 8–10 d after feeding on an infectious blood meal in summer and longer at winter temperatures. Based on conservative daily survival rates of 70–75% [87,88], it was estimated that the highest proportion of *Cx. annulirostris* surviving to 10 days was 0.08 in the summer months.

### 4.2. Population Dynamics

The impact of survival on arbovirus transmission can be offset by high mosquito populations, which are intimately linked to weather and other environmental variables. In northern Australia, populations of *Cx. annulirostris* are generally present throughout the year, although peaks in abundance vary within and between years, as well as between locations. In northern and southwestern Queensland, populations tended to peak during the mid-to-late wet season when there was an abundance of larval habitats [89]. This contrasts with the Northern Territory, where populations were at their highest at the end of the wet season (May–June) when constant inundation of larval habitats ceased and they became conducive to colonization by *Cx. annulirostris* [90].

In southern Australia, *Cx. annulirostris* populations peak in summer (December–February) before they decline and are not detected during winter months (June–August; [91,92]). Abundant *Cx. annulirostris* populations, which can exceed 9000 mosquitoes per trap in some locations, were one of the main drivers of the 1974 MVEV and 2011 WNV_KUN_ outbreaks in southeastern Australia [93,94]. In both these instances, above-average rainfall caused by La Niña weather systems provided widespread flooded habitats for the proliferation of *Cx. annulirostris* and waterbird breeding. Similarly, above-average rainfall associated with the 2021–2022 La Niña weather event led to a convergence of wading birds and mosquitoes, which, coupled with intensive pig farming, produced ideal conditions for JEV to emerge in southeastern Australia.

Whilst rainfall undoubtedly influences the population dynamics of these southern populations, they are also temperature dependent. It was suggested that average daily temperatures need to exceed 17.5 °C to sustain *Cx. annulirostris* populations [95] and when temperatures are below this, females overwinter as parous adults via quiescence [96]. Russell [91] suggested that infected parous *Cx. annulirostris* could overwinter viruses in southern Australia.

### 4.3. Dispersal

Mosquito dispersal can drive the movement of the virus within and between transmission foci and is a factor that requires consideration when formulating control strategies. In the Murray Valley of southeastern Australia, Russell [68] revealed that some *Cx. annulirostris* females can disperse at least 7 km from larval habitats whilst searching for a blood meal host. Subsequent mark–release–recapture experiments showed that the mean distance traveled by *Cx. annulirostris* ranged from 3.8 to 4.4 km from a release point, although a small proportion of marked females were able to travel over 12 km [97,98]. Thus, any disease mitigation strategies, such as larval control or relocation of domestic pigs away from productive larval habitats, may need to encompass these flight ranges, especially when mosquito populations are abundant [40,68].

Female *Cx. annulirostris* are also able to traverse hundreds of kilometers assisted by atmospheric wind transport, providing a mechanism for the long-range dispersal of viruses [99]. Kay and Farrow [100] used kites deployed in the planetary boundary layer (<100 m) to show that, whilst most flights were <150 km, it was estimated that on some nights, *Cx. annulirostris* had traveled up to 594 km. Similar long-range dispersal of *Cx. tritaeniorhynchus* was observed in Southeast Asia [101,102]. Using data from backtrack simulations, Ritchie and Rochester [17] suggested that infected *Cx. annulirostris* carried on low-level winds generated by low-pressure systems may have introduced JEV into the Torres Strait and onto Cape York Peninsula. Long-range mosquito-mediated virus dispersal is certainly plausible, as it is approximately 500 km from potential JEV foci in the New Guinea landmass to the Mitchell River area of Cape York Peninsula, the previous southern limit of the virus in Australia. However, it is over 2500 km from previously recognized foci in northern Australia and the southern limits of JEV reported during the 2022 outbreak. This would suggest that other mechanisms of virus dispersal were responsible, including infected migratory birds or overlapping mosquito–vertebrate cycles that utilize La Niña driven rainfall patterns.

### 4.4. Genetic Variation between Populations

Hemmerter et al. [103] revealed that five lineages of *Cx. annulirostris* occur in the Australasian region and suggested that these lineages may vary in their ability to transmit JEV. In particular, they noted that the ann-PNG1 and ann-PNG2 lineages shared a similar southern limit to JEV in northern Australia, hypothesizing that they may be efficient vectors of the genotype 1 JEV that replaced the genotype 2 viruses. Conversely, the ann AUS lineage present in all sampling sites on the Australian mainland and used for most vector competence experiments to date may not be as efficient a vector of the genotype 1 virus when compared with its high competence for the genotype 2 virus. The intensity and spread of the 2022 JEV epizootic in southeastern Australia suggest that the ann AUS and/or the ann S-AUS lineages, with the latter found only in southern locations, are highly competent vectors of the virus responsible.

The population genetics of the *Cx pipiens* complex, of which, *Cx. quinquefasciatus*, *Cx. australicus*, *Cx. molestus* and *Cx. globocoxitus* occur in Australia, is poorly understood. Recent analysis suggests that *Cx. australicus* and *Cx. globocoxitus* diverged from *Cx. quinquefasciatus* sometime after the latter species was introduced [104]. Preliminary evidence suggests that there is some genetic diversity in *Cx. quinquefasciatus* populations in Australia (N. Beebe, unpublished data), which may help to explain the difference in vector competence between the two Queensland populations examined.

### 4.5. Establishment of Exotic Vectors of JEV

Whilst *Cx. annulirostris* should be considered the primary vector of JEV, it is concerning that two major vectors of JEV from southeast Asia have become established in Australia in the last 20 years. *Cx gelidus* was first recognized in Brisbane in 1999 [105] and subsequently found in the Northern Territory [106], far north Queensland [107] and northern Western Australia (M Lindsay and A Broom, pers. comm. cited by Williams et al. [108]). Using climate suitability modeling, Williams et al. [108] concluded that much of Australia is suitable for the establishment of *Cx. gelidus*. The collection of a single specimen of *Cx. gelidus* in central New South Wales represents the southernmost record of this species in Australia (Webb, C.E., personal communication). On occasion, high numbers of *Cx. gelidus* larvae can be found in polluted water sites, such as sewage settlement ponds or run-off from animal processing plants [106]. However, only low numbers of *Cx. gelidus* are collected in CO_2_-baited light traps and Whelan et al. [106] suggested that these may not be the most efficient traps for sampling this species.

In 2020, specimens of *Cx. tritaeniorhynchus* were collected from the Darwin and Katherine regions of the Northern Territory [109]. This species was subsequently identified in northern Western Australia, suggesting that it is now widespread in northern Australia. So far, only low numbers of *Cx. tritaeniorhynchus* (<40 per trap) are collected relative to *Cx. annulirostris* (N. Kurucz, personal communication). Like *Cx. gelidus*, the collection of low numbers of *Cx. tritaeniorhynchus* may reflect the inefficiency of CO_2_-baited light traps for sampling this species. Regardless, as it expands its distribution, it may become more abundant as it finds more suitable conditions.

## 5. Directions for Further Investigation

The original incursions of genotype 1 and 2 viruses into northern Australia led to a considerable amount of research into the role that Australian mosquito species play in transmission cycles. However, much of the work centered on locations in northern Australia where the virus was active or that possessed ecological conditions conducive to virus transmission. In this section, we identify directions for the investigation that will inform our knowledge of the role that Australian mosquitoes could play in the transmission of the emergent virus genotype and how intrinsic and ecological factors could influence transmission dynamics.

### 5.1. Detection of the Virus in Mosquito Field Populations during Periods of Recognized Virus Activity

These investigations should focus on processing pools according to taxonomic groups so that infection rates in different species can be compared to help incriminate vectors. Collections should be conducted within and between expected transmission seasons to provide information on the temporal and spatial distribution of the virus, as well as its ability to overwinter. Alternatives to the standard CO_2_-baited light traps used by most jurisdictions should also be utilized, including gravid traps, which collect *Cx. quinquefasciatus* and, potentially, *Cx. gelidus*.

### 5.2. Vector Competence of Australian Mosquito Species for the Newly Emergent JEV Genotype

Representative populations from different latitudes of Australia should be included to determine whether there are within and between species differences in their ability to become infected with and transmit the virus. Once this has been established, the population genetics of key JEV vectors that can influence transmission dynamics should be determined. For instance, it is possible that the southern lineage of *Cx. annulirostris* could be more efficient vectors of the virus genotype that has emerged in southern Australia. Similar mosquito genotype X virus genotype interactions were proposed for dengue viruses in *Aedes aegypti* [110].

### 5.3. The Genetic Diversity of Implicated Vectors

Clearly, there is a considerable amount of genetic diversity within the *Cx. sitiens* subgroup, particularly within *Cx. annulirostris* and *Cx. palpalis*. The population genetics of *Cx. quinquefasciatus* and other members of the *Cx. pipiens* complex is even less well known. Thus, widespread sampling of implicated vectors needs to be undertaken so that the genetic structure of these mosquitoes can be determined and the link to bionomics and vector competence established.

### 5.4. The Potential for JEV to Overwinter in Australian Mosquitoes

Laboratory-based experiments should be undertaken to establish potential filial transmission rates in common floodwater *Aedes* of southern Australia, such as *Ae. vittiger*, *Ae. sagax* and *Ae. theobaldi*. Detection of the virus in field-collected *Aedes* spp. males or adults reared from larvae will provide evidence of vertical transmission in nature. Although rarely documented in temperate JEV endemic areas of Southeast Asia [111,112], infected overwintering parous *Culex* females provide another possible mechanism of virus maintenance that should be investigated.

### 5.5. Bionomics of Invasive Australian JEV Vectors

The geographical distribution of *Cx. gelidus* and *Cx. tritaeniorhynchus* in Australia needs to be defined, along with their population dynamics, preferred larval habitats and biting behavior.

### 5.6. Host-Feeding Patterns of Incriminated Vectors

These studies need to ascertain the feeding rates on pigs and birds and should focus on mosquitoes found in proximity to intensive pig farms, locations where feral pigs congregate, and ardeid and other waterbird colonies. Feeding on macropods in southeastern Australia also needs to be investigated to determine whether there is a virus dilution effect like that suggested in northern areas. Where possible, vertebrate surveys should be undertaken so that the influence of host abundance on mosquito feeding preference can be quantified [113]. Of particular interest would be the proportion of implicated vectors feeding on domesticated versus feral pigs.

### 5.7. Australian Vertebrate Fauna as Amplifying Hosts of JEV

There is only limited information on the JEV viremia levels in Australian vertebrates. Experimental inoculation of two ardeid birds, namely, *N. caledonicus* and intermediate egrets (*Egretta intermedia*), and brushtail possums (*Trichosurus vulpecula*) produced moderate viremia levels [66,114]. The latter study also demonstrated that macropods produced only low-level viremia when inoculated with JEV [66]. However, it is difficult to interpret these results, as these viremias may still have been sufficient to infect efficient mosquito vectors. Thus, vertebrate infection studies should be conducted using infected mosquitoes as the source of infection and uninfected recipient mosquitoes used to measure whether candidate vertebrate species are infectious post-exposure. This would show the correlation between vertebrate viremia levels and mosquito infection, and provide a more accurate representation of what would be encountered in the field than if needle inoculation is used for infection and only the viremia levels are measured.

## 6. Conclusions

Soon after JEV emerged in the mid-1990s, it was postulated that environmental conditions were suitable for the establishment of the virus on the Australian mainland [22]. However, despite at least two introductions of the virus into the region [13,20], there was no evidence to suggest this scenario eventuated [19]. That is what makes the emergence of JEV in southeastern Australia, at least 2500 km below its previous southern geographical limit, such an unanticipated event.

Given the geographical extent of virus activity and evidence the virus was present prior to the widespread 2021–2022 outbreak, it is unlikely that the virus will be eliminated from Australia. Other human and animal health management strategies will have to be implemented, including vaccination, changes in animal husbandry and mosquito control. The synthesis of current knowledge of JEV vectors presented herein reinforces that it is *Culex* spp., particularly *Cx. annulirostris*, that will need to be the target of surveillance and control activities. Any programs will need to be sustainable and flexible enough to incorporate data that will arise from investigations conducted to characterize the role that endemic and newly established mosquitoes play in the transmission of JEV in Australia.

## Figures and Tables

**Table 1:** Detection of the Japanese encephalitis virus or viral RNA in mosquitoes collected from northern Australia and Papua New Guinea, 1995–2005.

Location	Year	Number of *Culex* Processed	Number of Mosquitoes of Other Genera Processed	Trapping Method ^a^	Number of JEV Positive Pools	Composition of Positive Pools	Reference
Badu (Torres Strait)	1995	2987	10,313	CDC-LT	8	*Culex sitiens* subgroup ^b^	[37]
	1998	26,158	5730	CDC-LT	42	*Cx. sitiens* subgroup	[38]
				CDC-LT	1	*Aedes vigilax*	
	2000	7779	83,461	CDC-LT	1	*Culex gelidus*	[39]
	2002	Not separated by taxonomic group ^c^		MM	3	Unknown	[24]
	2003	22,157	Not processed ^d^	CDC-LT, MM	18	*Cx. sitiens* subgroup	[24,40]
				CDC-LT, MM	2	*Cx. gelidus*	[24], this paper
				CDC-GT	1	*Culex bitaeniorhynchus*	This paper
	2004	1795	Not processed	NMT	1	*Cx. gelidus*	[24]
	2005	4563	Not processed	NMT	1	*Cx. sitiens* subgroup	[24]
Moa (Torres Strait)	2003	1400	Not processed	MM	2	*Cx. sitiens* subgroup	[24]
				MM	1	*Cx. gelidus*	[24]
Cape York Peninsula	2004	23,144	Not processed	CDC-LT	1	*Cx. sitiens* subgroup	[21]
Saibai (Torres Strait)	2000	45,581	38,473	CDC-LT	1	*Cx. sitiens* subgroup	[41]
Papua New Guinea	1997	35,038	25,445	CDC-LT	1	*Cx. sitiens* subgroup	[15]
	1998	210,200	49,717	CDC-LT	2	*Cx. sitiens* subgroup	

^a^ Trap used to collect mosquitoes for virus detection: CDC-LT is Centers for Disease Control light trap baited with CO_2_ with or without 1-octen-3-ol; MM is “Mosquito Magnet^®^”, a propane-powered updraft trap; CDC-GT is Centers for Disease Control gravid trap; and NMT is the Northern Australia Quarantine Strategy Mozzie Trap, which is a CO_2_-baited updraft trap. ^b^ Includes the morphologically similar species *Culex annulirostris*, *Cx. palpalis* and *Cx. sitiens*. ^c^ These mosquitoes were collected as part of a trial of a remote trapping system and were processed without being sorted and processed according to the taxonomic group. ^d^ After 2000, only members of the *Culex* genus were processed.

## Data Availability

Not applicable.

## Supplementary Materials

The following supporting information can be downloaded from https://www.mdpi.com/article/10.3390/v14061208/s1. Table S1: Mosquitoes collected from the Torres Strait islands of Badu and Saibai, northern Australia, and the Western Province of Papua New Guinea between 1995 and 2000, and processed for the detection of Japanese encephalitis virus.
