# Supplementary material for: The Emergence of Japanese Encephalitis Virus in Australia in 2022: Existing Knowledge of Mosquito Vectors
Source: Viruses. 2022 Jun 2;14(6):1208. doi: 10.3390/v14061208 (PMC9231386; doi:10.3390/v14061208)
Supplement: Supplementary file 1 [file viruses-14-01208-s001.zip › viruses-1750044-supplementary.pdf]

Supplementary Table S1. Mosquitoes collected from Torres Strait islands of Badu and Saibai, northern Australia, and the Western Province of Papua New Guinea between 1995 and 2000, and processed for detection of Japanese encephalitis virus (from [1–5]).

	**Collection location**		
**Species**	**Badu**	**Saibai**	**Papua New Guinea**
Aedeomyia catasticta	0	0	178
Aedes aurantius aurantius	0	3	179
Ae. culiciformis	24,839	0	0
Ae. iwi	7	0	0
Ae. katherinensis	2	0	0
Ae. kochi	50,612	2,688	2,388
Ae. littlechildi	453	1	6
Ae. notoscriptus	33	0	9
Ae. scutellaris	139	15	0
Ae. stoneorum	10,313	0	20
Ae. tremulus	54	0	0
Ae. vigilax	3,249	601	1,721
Anopheles bancroftii	0	0	601
An. hilli	28	6	0
Anopheles farauti sensu lato[a]	136	3,446	29,262
Anopheles spp.[b]	0	1	0
Armigeres papuensis	0	0	257
Bironella simmondsi	0	0	98
Coquillettidia crassipes	2	15,787	1,139
Culex australicus	0	0	186
Cx. bitaeniorhynchus	24	0	1,212
Cx. gelidus	84	93	0
Cx. hilli	0	0	296
Cx. pullus	0	0	87
Cx. quinquefasciatus	4	733	1598
Cx. sitiens	3	0	0
Cx. sitiens subgroup[b]	35,815	44,755	219,520
Cx. starkeae	106	0	0
Cx. vicinus	866	0	22,339
Cx. whitmorei	22	0	0
Ficalbia minima	0	0	535
Mansonia papuensis	0	0	1,992
Mansonia septempunctata	0	0	1,044
Ma. uniformis	0	428	1,636
Tripteroides alboscutellatus	0	0	6
Tp. atripes	18	1	3

Tp. magnesianus	1	0	1
Uranotaenia albescens	2	5	0
Uranotaenia spp.	1	26	0
Verrallina carmenti	8,909	0	602
Ve. funerea	706	15,465	30,065
Ve. lineata	0	0	2,334
Verrallina spp.	0	0	1,086
Total processed	136,428	84,054	320,400

[a]*Anopheles farauti sensu lato* refers the members of the *Anopheles farauti* species complex [6].
[b]Genus names followed by "spp." represents specimens that could not be identified to species level.
[c]Includes the morphologically similar species *Culex annulirostris*, *Cx. palpalis* and *Cx. sitiens*.

1. Johansen, C.A.; Nisbet, D.J.; Foley, P.N.; van den Hurk, A.F.; Hall, R.A.; Mackenzie, J.S.; Ritchie, S.A. Flavivirus isolations from mosquitoes collected from Saibai Island in the Torres Strait, Australia, during an incursion of Japanese encephalitis virus. *Med Vet Entomol* **2004**, *18*, 281-287, doi: 10.1111/j.0269-283X.2004.00510.x.
2. Johansen, C.A.; van den Hurk, A.F.; Pyke, A.T.; Zborowski, P.; Phillips, D.A.; Mackenzie, J.S.; Ritchie, J.S. Entomological Investigations of an outbreak of Japanese encephalaitis virus in the Torres Strait, Australia, in 1998. *J Med Entomol* **2001**, *38*, 581-588, doi: 10.1603/0022-2585-38.4.581.
3. Johansen, C.A.; van den Hurk, A.F.; Ritchie, S.A.; Zborowski, P.; Paru, R.; Bockarie, M.J.; Drew, A.C.; Khromykh, T.I.; Mackenzie, J.S. Isolation of Japanese encephalitis virus from mosquitoes (Diptera: Culicidae) collected in the Western Province of Papua New Guinea, 1997-1998. *Am J Trop Med Hyg* **2000**, *62*, 631-63, doi: 10.4269/ajtmh.2000.62.631.
4. Ritchie, S.A.; Phillips, D.; Broom, A.; Mackenzie, J.; Poidinger, M.; van den Hurk, A. Isolation of Japanese encephalitis virus from *Culex annulirostris* in Australia. *Am J Trop Med Hyg* **1997**, *56*, 80-84, doi: 10.4269/ajtmh.1997.56.80.
5. van den Hurk, A.F.; Nisbet, D.J.; Johansen, C.A.; Foley, P.N.; Ritchie, S.A.; Mackenzie, J.S. Japanese encephalitis on Badu Island, Australia: the first isolation of Japanese encephalitis virus from *Culex gelidus* in the Australasian region and the role of mosquito host-feeding patterns in virus transmission cycles. *Trans Royal Soc Trop Med Hyg* **2001**, *95*, 595-60, doi: 10.1016/s0035-9203(01)90090-2.0.
6. Beebe, N.W.; Russell, T.; Burkot, T.R.; Cooper, R.D. *Anopheles punctulatus* group: evolution, distribution, and control. *Ann Rev Entomol* **2015**, *60*, 335-350, doi:10.1146/annurev-ento-010814-021206.
